# Supplementary material for: Leveraging the Structure of DNAJA1 to Discover Novel Potential Pancreatic Cancer Therapies
Source: Biomolecules. 2022 Sep 29;12(10):1391. doi: 10.3390/biom12101391 (PMC9599757; doi:10.3390/biom12101391)
Supplement: Supplementary file 1 [file biomolecules-12-01391-s001.zip › biomolecules-1897947-supplementary.pdf]

Supplemental Information

for

Leveraging the Structure of DNAJA1 to Discover Potentially Novel Pancreatic Cancer Therapies

Heidi E. Roth,[1] Aline de Lima Leite,[1,2] Nick Palermo,[3] and Robert Powers[1,2*]

[1]*Department of Chemistry, University of Nebraska-Lincoln, Lincoln NE 68588-0304*

[2]*Nebraska Center for Integrated Biomolecular Communication, University of Nebraska-Lincoln, Lincoln NE 68588-0304*

[3]*Holland Computing Center, University of Nebraska-Lincoln, Lincoln, NE 68588-0150*

*To whom correspondence should be addressed:

Robert Powers
University of Nebraska-Lincoln
Department of Chemistry
722 Hamilton Hall
Lincoln, NE 68588-0304
Email: rpowers3@unl.edu
Phone: (402) 472-3039
Fax: (402) 472-9402

Keywords: DNAJA1, Molegro Virtual Docker, drug discovery, Hsp40

Table of Contents

Figure S1. DNAJA1-107 amino acid sequence.

Figure S2. Structures and nomenclatures of lead compounds.

Figure S3. Binding of 7727968 to DNAJA1-107.

Figure S4. Binding of 910120 to DNAJA1-107.

Table S1. Phosphorous compounds used to calibrate ligand efficiency (LE).

Table S2. Pairwise Tanimoto coefficients for lead compounds.

MVKETTYYDVLGVKPNATQEELKKAYRKLALKYHPDKNPNEGEKFKQISQA
YEVLSDKRLGGEQAIKEGGAGGGFGSPMDIFDMFFGGGGRMQRERRGKNV

Figure S1. DNAJA1-107 amino acid sequence. One letter amino acid code for the first 107 residues of DNAJA1. Residues in red denote missing assignments in the NMR data.

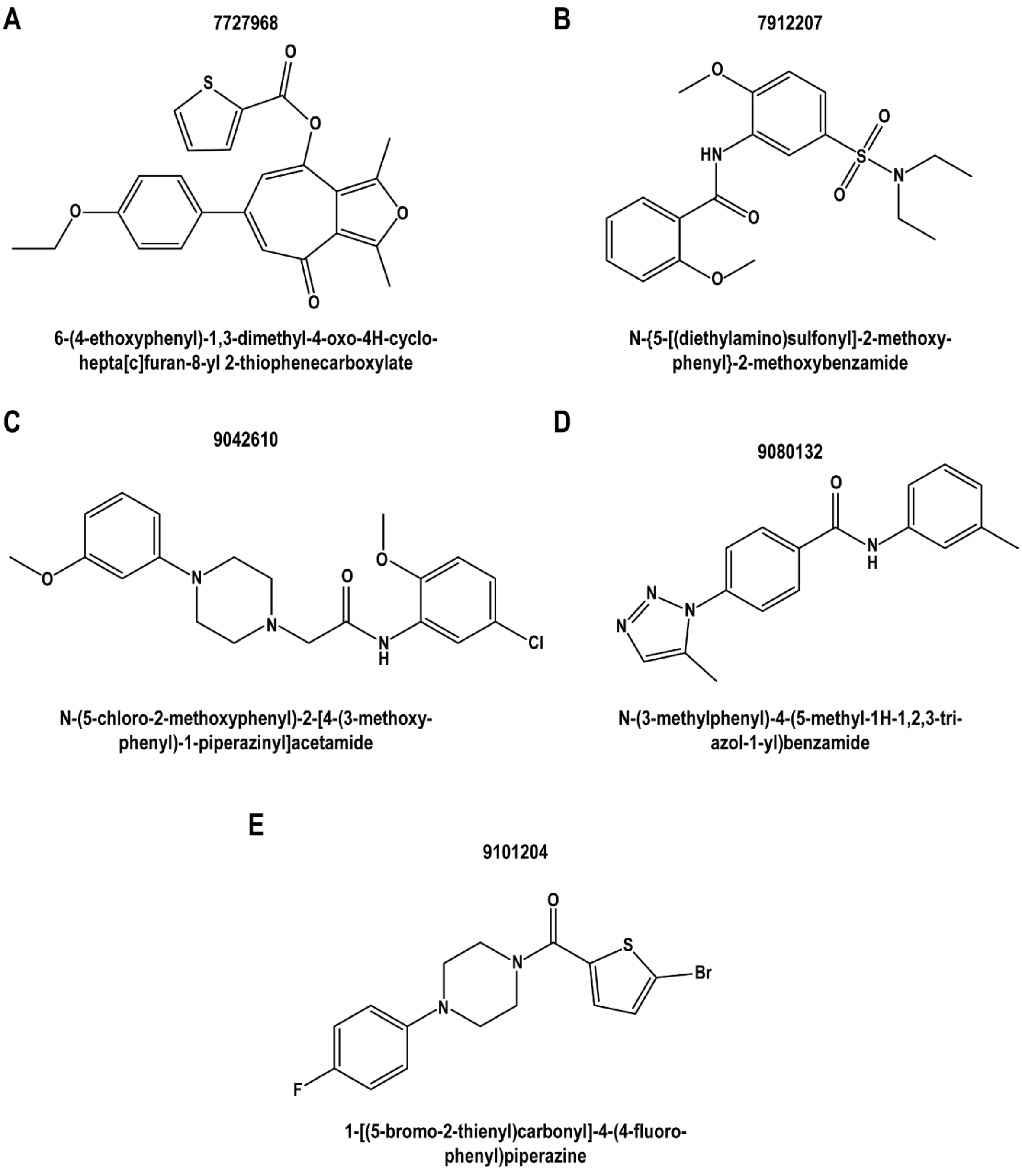

Figure S2. Structures and nomenclatures of lead compounds. Compounds confirmed to bind DNAJA1-107 were ordered from Hit2Lead.com© and include **A.** 7727958, **B.** 7912207, **C.** 9042610, **D.** 9080132, and **E.** 9101204.

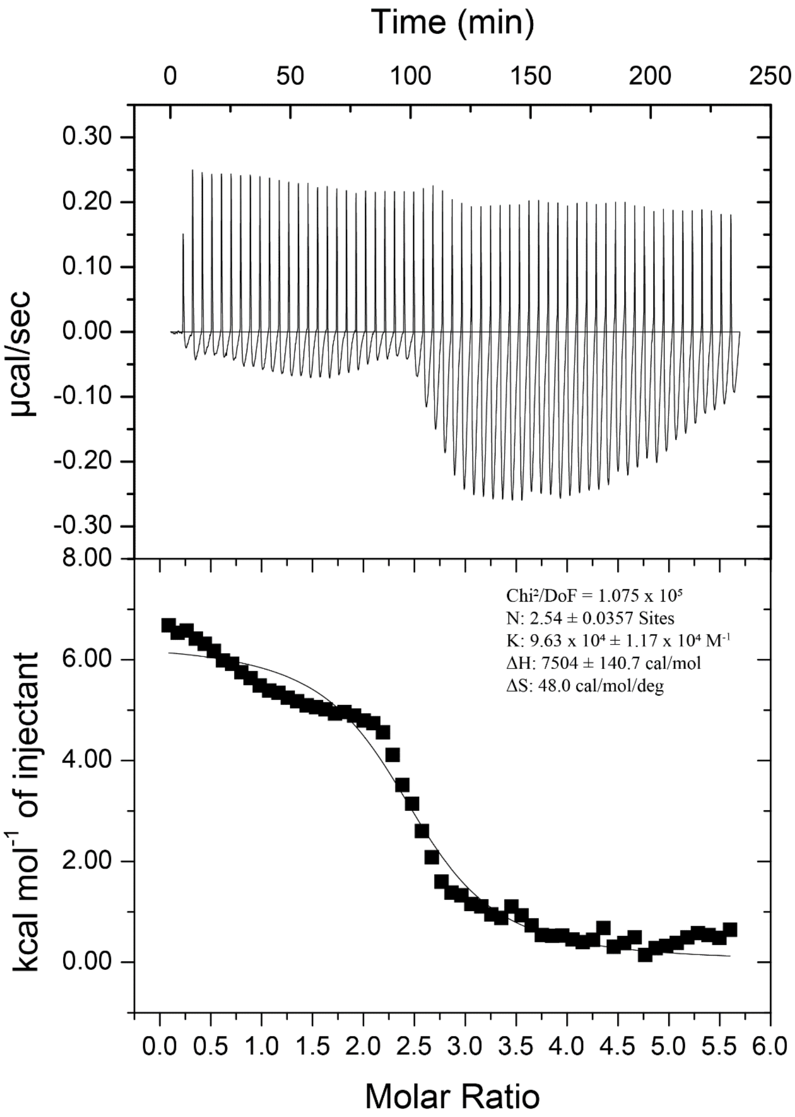

Figure S3. Binding of 7727968 to DNAJA1-107. Isothermal titration calorimetry (ITC) plot demonstrating the binding of 7727968 to DNAJA1-107. (*top*) Raw isothermal ITC data corresponding to the heat intensity versus injection number and plotted as equivalent ratios. (*bottom*) Plot of the integrated enthalpies normalized per mol of injectant and normalized against the equivalent ratios. The raw data was fit to a standard binding curve with stoichiometry set as a variable. The best-fit line is superimposed onto the experimental data and the resulting thermodynamic parameters are shown as an insert.

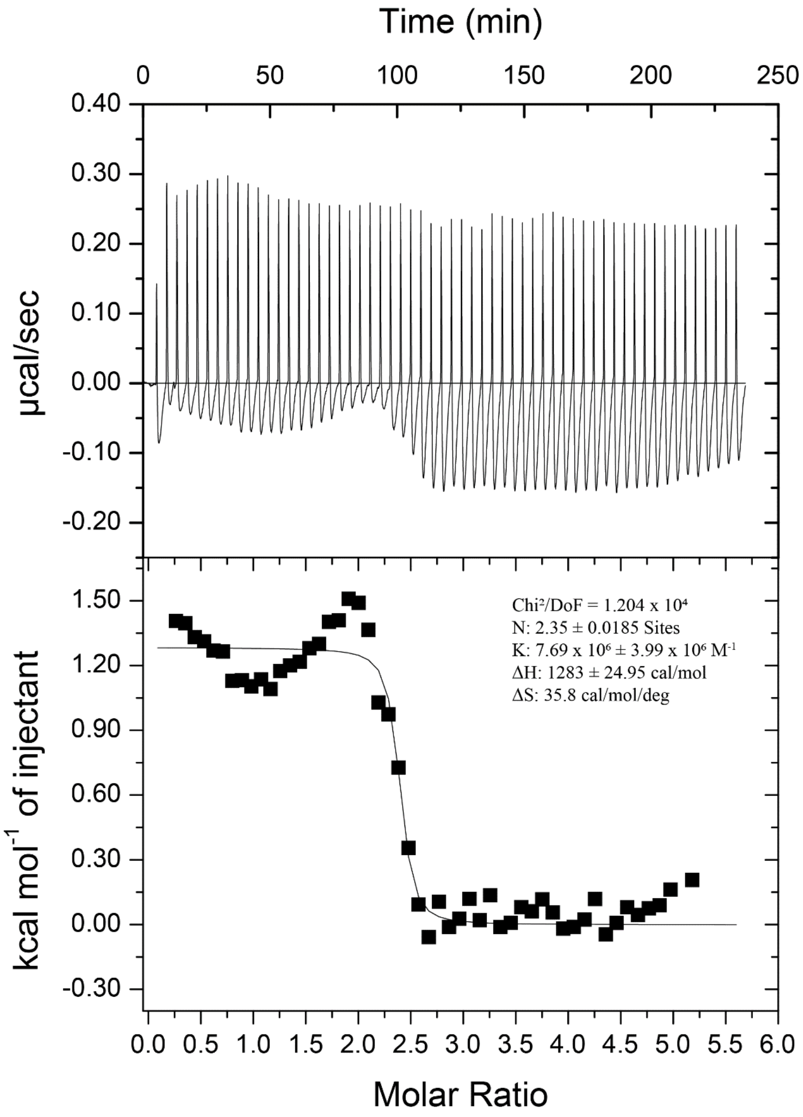

Figure S4. Binding of 910120 to DNAJA1-107. Isothermal titration calorimetry (ITC) plot demonstrating the binding of 910120 to DNAJA1-107. (*top*) Raw isothermal ITC data corresponding to the heat intensity versus injection number and plotted as equivalent ratios. (*bottom*) Plot of the integrated enthalpies normalized per mol of injectant and normalized against the equivalent ratios. The raw data was fit to a standard binding curve with stoichiometry set as a variable. The best-fit line is superimposed onto the experimental data and the resulting thermodynamic parameters are shown as an insert.

Table S1. Phosphorous compounds used to calibrate ligand efficiency (LE)
6 -Phosphogluconic acid trisodium salt
Adenosine 5'-diphosphate sodium salt
alpha-D-glucose 1,6-phosphate disodium salt hydrate
Dihydroxyacetone phosphate hemimagnesium salt hydrate
D-fructose 1,6-bisphosphate trisodium salt hydrate
D-fructose 6-phosphate disodium salt hydrate
D-Mannose 6-phosphate disodium salt hydrate
D-Ribose 5-phosphate disodium salt dihydrate
Glycerol 2-phosphate disodium salt hydrate
Lithium potassium acetyl phosphate
O-phospho-L-serine
O-phospho-L-Tyrosine
O-Sulfo-L-serine
rac -Glycerol 3 -phosphate disodium salt
Riboflavin 5'-phosphate
Thiamine pyrophosphate

Table S2. Pairwise Tanimoto coefficients for lead compounds[a,b]

	**7727968**	**7912207**	**9042610**	**9080132**	**9101204**
**7727968**	1	0.30 (0.16)	0.16(0.16)	0.22 (0.16)	0.14 (0.16)
**7912207**	0.30 (0.16)	1	0.23(0.29)	0.28 (0.44)	0.14 (0.23)
**9042610**	0.16(0.16)	0.23(0.29)	1	0.20(0.26)	0.25(0.41)
**9080132**	0.22 (0.16)	0.28 (0.44)	0.20(0.26)	1	0.15 (0.26)
**9101204**	0.14 (0.16)	0.14 (0.23)	0.25(0.41)	0.15 (0.26)	1

[a]Values outside parentheses represent atom pair (AP) coefficients. Values in parentheses represent maximum common substructure (MCS) coefficients.
[b]See **Figure S2** for the compound IDs and structures
